# Preparation of pH Sensitive Pluronic-Docetaxel Conjugate Micelles to Balance the Stability and Controlled Release Issues

**DOI:** 10.3390/ma8020379

**Published:** 2015-01-23

**Authors:** Yanchao Liang, Zhihui Su, Yao Yao, Na Zhang

**Affiliations:** School of Pharmaceutical Science, Shandong University, 44 Wenhua Xi Road, Ji’nan 250012, Shandong, China; E-Mails: liangyanchao2012@126.com (Y.L.); suzhihui2013@163.com (Z.S.); yaoyao2y@126.com (Y.Y.)

**Keywords:** docetaxel, conjugates, nano-micelles, stability, pH controlled release

## Abstract

A novel polymer-drug conjugate was prepared by the chemical reaction between the copolymer Pluronic P123 and the docetaxel via a pH sensitive hydrazone bond. These pluronic P123-docetaxel (DTX) conjugates (P123-DTX) could form the stable drug-loaded materials that can self-assemble into the defined nano-micelles in aqueous solution because of their obvious amphiphilic property and low critical micelle concentration. The spherical morphology and particle size of the prepared nano-micelles were characterized by transmission electron microscopy and dynamic light scattering, respectively. Moreover, after the introduction of pH sensitive hydrazone bond, P123-DTX micelle showed a pH dependent drug release behavior. At pH 5.0 (in 48 h), the cumulative release amount of DTX were ~84.9%, which is about six times higher than that at pH 7.4. The prepared novel p123-DTX conjugates may offer a great benefit for drug delivery and controlling the drug release.

## 1. Introduction

One purpose to design smart materials is for the sake of its better applications in the field of medicine [1]. Recently, some novel systems designed by smart polymers have been frequently used as drug delivery devices [2,3,4]. Among these polymeric systems, micelles formed by amphiphilic copolymers had received more attentions. Composed of two distinct blocks, these copolymers could self-assemble to form two distinct domains in aqueous solution: a hydrophobic core and a hydrophilic shell [5]. In this regard, hydrophobic drugs such as anticancer drugs can be entrapped into the hydrophobic core and sheltered by the hydrophilic shell. These covered drug will achieved an improved water solubility capacity and be protected from enzymatic degradation and uptake by mononuclear phagocytes, macrophages and reticuloendothelial systems in the liver, spleen and bone marrow. Hence, their blood circulation time prolonged. More than that, for anticancer drugs, the formed micelles with nano-size will also have the advantages to avoiding the premature elimination via glomerular filtration in the kidneys and stay within the tumor tissues due to the enhanced permeation and retention (EPR) effect [6]. The past decade has witnessed the development of the polymeric micelles and several types of anticancer drug loaded micelles have been applied in hospital or investigated clinical trials [7]. Micelles formed by copolymers have shown its huge potential in anticancer drug delivery.

However, these self-assembled micellar carriers often expose inadequate *in vivo* stability, which leads to premature drug release following intravenous injection. In this respect, polymeric micelles formed by conjugating drugs to the polymer backbone via covalent bonds can be an attractive strategy, which also known as polymer therapeutics, to eliminate the burst and premature release of the drug [8]. The covalent bonds in the polymer-drug conjugates can hold the drug firmly in the circulation, and there will be less anticancer drug released into healthy tissue [9]. In the past several years, various types of polymer-drug conjugates have been explored to enhance the *in vivo* stability of drug-loaded biodegradable micelles. And many of them have been evaluated in clinical trials [10]. While the release profile of the polymer-drug conjugates still need to be optimized to ensure the therapeutic efficacy. In fact, although a lot of work in the research of the polymer-drug conjugates had showed a superior stability against dilution, these micelles may also adversely influence drug release in the tumor tissues and cells, leading to compromised therapeutic outcomes.

To resolve the extracellular stability *versus* intracellular drug release dilemma, many researchers have dedicated their study to designing the bio-responsive (such as pH and enzyme-sensitive) micelles [11]. In particular, pH-responsive micelles have attracted great attention due to the existence of mildly acidic pH in the tumor tissues than the normal tissues, which may provide a tissues-specific stimulus that can be exploited for selective drug release [12]. In the construction of the pH sensitive conjugates, a range of chemical bonds (orthoester, acetal, hydrazone, imine, cis-aconytil and trityl bonds included) have been developed to show an enhanced degradation or hydrolysis in the presence of slightly acidic media, while being stable at neutral pH level. It is noted that many of the researchers had focused their research on the study of hydrazone group, which had shown favorable pH-responsible ability in antitumor drug delivery [13,14,15]. Recently, a novel acid-cleavable prodrug of doxorubicin (INNO-206) were reported to be under phase II clinical development [16]. Binding with albumin via the acid-sensitive hydrazone contained linker, the INNO-206 conjugates can release doxorubicin selectively at the tumor site, which showed significantly superior antitumor efficacy and a good safety profile over free doxorubicin [17]. At this stage, the INNO-206 conjugates is evaluated for its effective antitumor therapy against different tumor xenograft models and has the potential to be a promising clinical candidate for treating a broad range of solid tumors [18]. Therefore, developing the polymeric micelles based on pH-responsive polymer-anticancer drug conjugates can be a potential way to improve the stability and drug release issue.

Similar with doxorubicin, docetaxel (DTX) is another one of the most potent chemotherapeutic agents, which exhibits a more effective antitumor activity than paclitaxel. Docetaxel has shown a broad spectrum of activity against a variety of tumors, especially for the treatment of breast, gastric, ovarian, prostate and non-benign lung cancer [19]. In our previous work, conjugates micelles formed by the triblock copolymer Poly (ethylene oxide)-block-poly (propylene oxide)-block-poly (ethylene oxide) (Pluronic) have shown their superior stability capacity in DTX delivery. Without immunogenicity, antigenicity or toxicity, these commercially available Pluronic copolymers can spontaneously form nanosized polymeric micelles in aqueous solution, which is benefit for DTX protection in the circulation. Besides, the effect of Pluronic copolymers in promoting active membrane transport and reversing the multidrug resistance (MDR) effect, may also be helpful to enhance the anticancer therapeutic effect. However, the release profile of these ester bonded conjugates are still suppressed and need to be optimized. Herein, the hydrazone group contained pH sensitive strategy can be utilized to improve their release properties while maintaining their stability in DTX delivery.

Under the above considerations, it is important to seek feasible hydrazone linkage construction method to prepare the pH sensitive conjugates. In this study, we chose the widely used triblock copolymer Pluronic P123 (P123) as the carrier to construct our polymer-docetaxel conjugates (P123-DTX). To achieve the pH-dependent characteristic, the hydrazone contained derivative of DTX was prepared via a two-step reaction, so that the hydrazone linker was formed before their combination with the carriers. The micelles formed by these P123-DTX conjugates were also evaluated and investigated to verify its potential to balance the stability and drug release.

## 2. Results and Discussion

**Figure 1:**
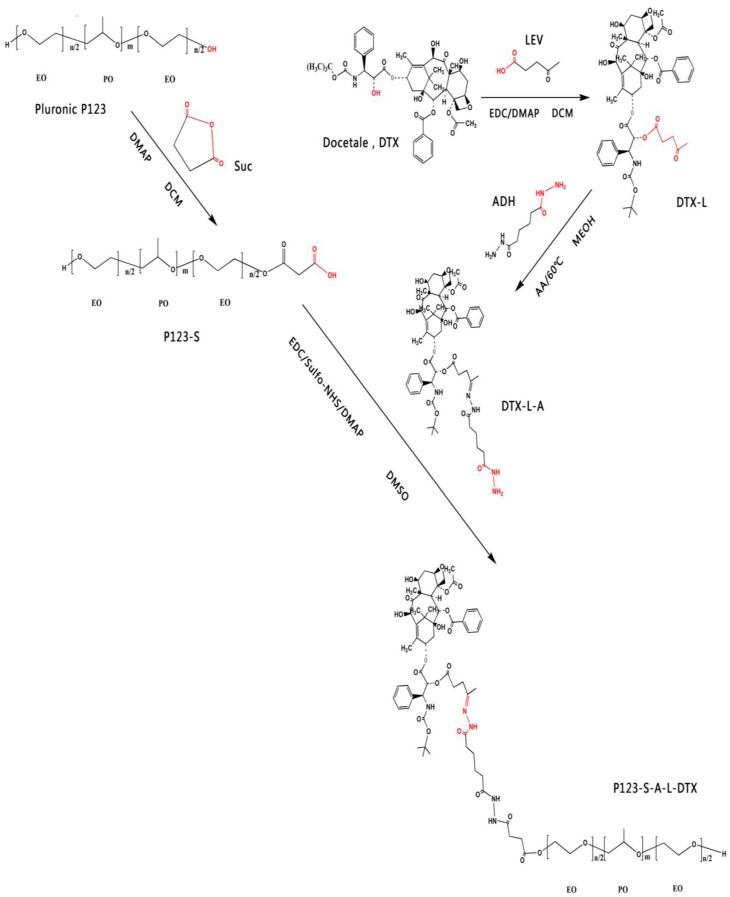
Synthetic routes used for the preparation of Pluronic P123-DTX.

The structure and synthesis steps the P123-DTX conjugates is illustrated in Figure 1. The pH sensitive Pluronic P123-DTX conjugates were prepared by the attachment between the DTX and the Pluronic P123.

**Figure 2:**
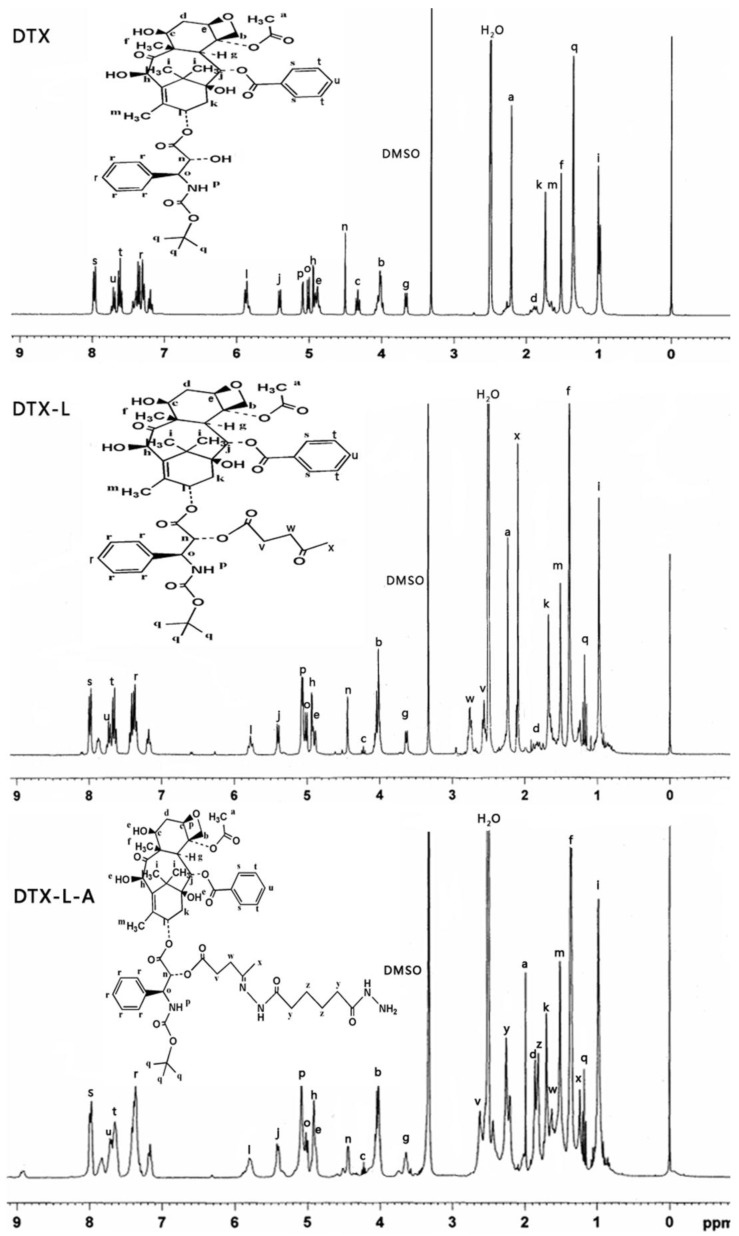
^1^H NMR spectrum of the derivatives of docetaxel (DTX) (DTX, DTX-L, DTX-L-A) in DMSO-*d*_6_.

**Figure 3:**
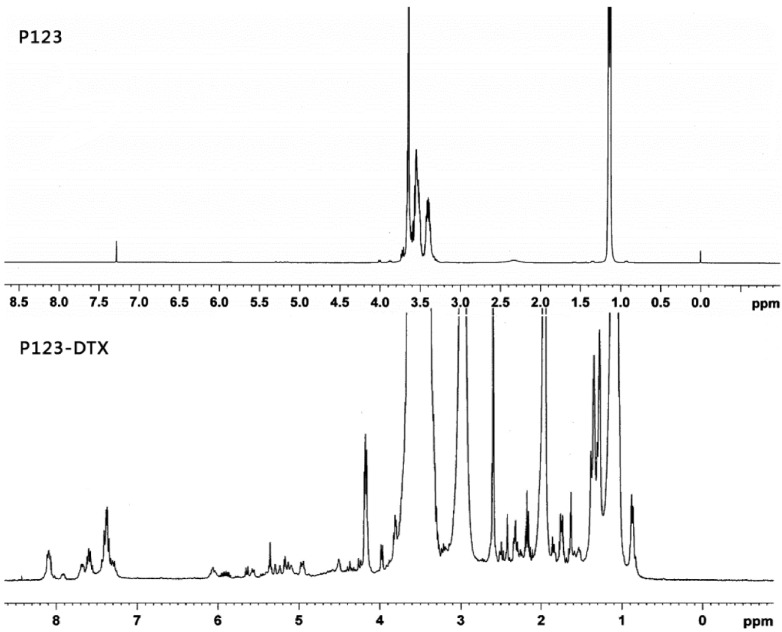
^1^H NMR spectrum of Pluronic P123 and P123-DTX conjugates in DMSO-*d*_6_.

Before the combination with the Pluronic P123, DTX was chemically modified to form the linker contained derivatives, which is important to exhibit its pH sensitive behavior. It had been reported that the modification of taxanes by levulic acid could be an effective way in forming the pH sensitive conjugate to fulfill the issue of stability and controlled release [20,21,22]. Therefore, the derivatives of the DTX was synthesized: (1) modification of DTX with a levulic acid (DTX-L); (2) synthesized the hydrazone bond contained derivative of DTX (DTX-L-A) by coupling the levulic acid-modified DTX with adipic dihydrazide. To be better conjugated, the carriers were also modified to achieve the Pluronic P123 derivatives: the succinic acid-modified Pluronic P123 (P123-S). The derivatives of DTX and Pluronic P123-DTX conjugates were characterized by ^1^H NMR, respectively (Figure 2 and Figure 3).

Based on the dialysis method [23], the P123-DTX conjugates can be self-assembled into nano-sized polymeric micelles because of their amphiphilic properties. After the preparation of the conjugates, their drug-loading efficiency was characterized. Based on our previous work [24], the weight percentage (wt%) of DTX in the P123-DTX conjugate was determined using the UV-Visible spectrophotometer at 230 nm using acetonitrile as solvent. The DTX contents of P123-DTX conjugates were determined to be ~11.18% (wt%).

**Figure 4:**
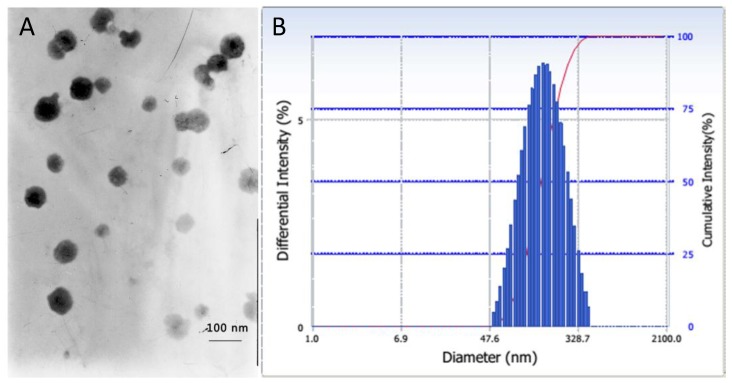
Characterization of P123–DTX conjugate micelles: (**A**) TEM image and (**B**) particle size by DLS.

The particle size is another crucial parameter for drug delivery, which could dramatically affect the *in vivo* pharmacokinetics and distributions of the conjugate micelles [25]. The particle size and size distribution of P123-DTX conjugate micelles were measured by dynamic light scattering (DLS) and their morphology were visualized by transmission electron microscopy (TEM) (Figure 4). These P123-DTX conjugate micelles were well dispersed as individual particles with spherical shape and showed an average diameter about 138 nm. With the limiting size less than 200 nm, these conjugate micelles could beneficial for more drug accumulation in solid tumors due to Enhanced Permeation and Retention effect (EPR effect) [26].

**Figure 5:**
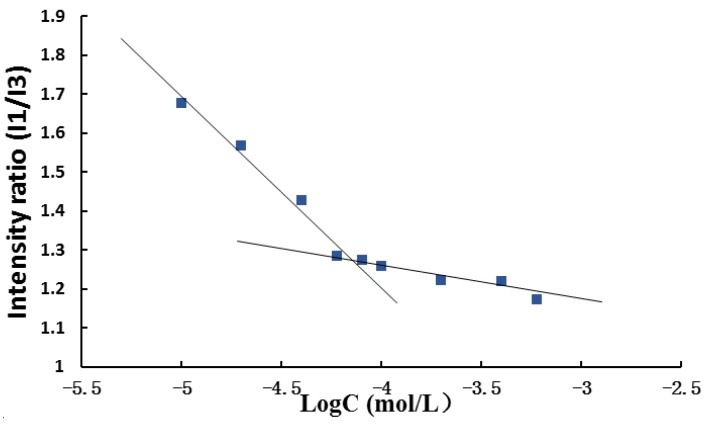
Critical micelle concentration (CMC) measurement using pyrene as fluorescent probe: plots of the intensity ratio (*I*_1_/*I*_3_) from pyrene emission spectra *versus* the log of concentration for pH sensitive P123-DTX conjugate ([pyrene] = 1.0 × 10^−7^ M).

The hydrophobic aggregation ability played an important role to form the micelles and protect the hydrophobic drugs from the cleavage of the system. In this respect, the critical micelle concentrations (CMC) of these conjugates could be used as the fundamental parameter to characterize the thermodynamic stability of micelles (Figure 5). The critical micelle concentration (CMC) of the P123-DTX conjugates was determined using the fluorescence probe technique as we described previously [24]. The fluorescence intensity ratios of pyrene at 373 and 383 nm (*I*_373_/*I*_383_, *I*_1_/*I*_3_) were calculated and plotted against the concentration logarithm of the P123-DTX conjugates. It could be seen in Figure 5, as the polymer concentration increased above the CMC, the *I*_1_/*I*_3_ value experienced a rapid decline process, because the probe pyrene was incorporated into the hydrophobic core of the micelles. The CMC was determined by taking the inflection point of the pyrene *I*_1_/*I*_3_ ratio plot. The measured CMC value of the P123-S-A-L-DTX conjugates was ~7.232 × 10^−5^ mol/L (~502.2 µg/mL). It also indicated that the Pluronic P123-DTX conjugates could form micelles in lower concentration, which would be helpful to form the stable micelles.

**Figure 6:**
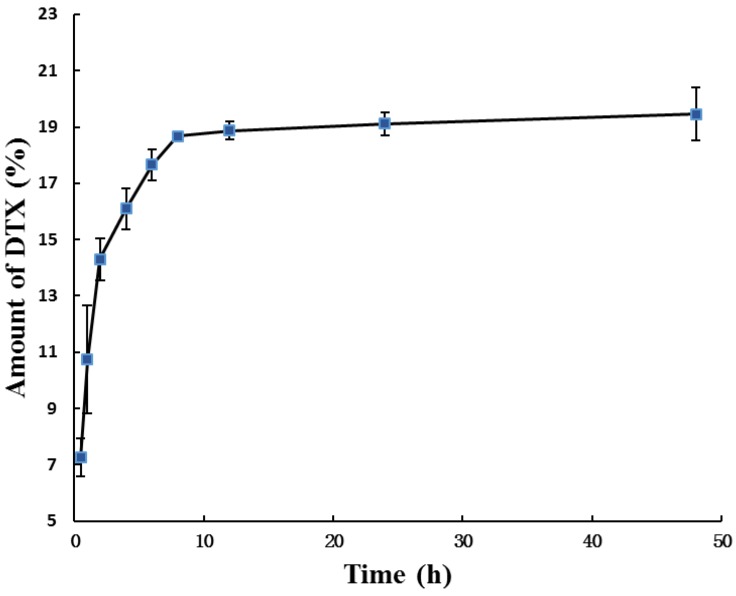
Stability of P123-DTX conjugate micelles following incubation in mouse plasma in at 37 °C for different times. The amount of DTX was calculated as the sum of DTX and DTX-L by HPLC method. Data are expressed as means ± SD (Standard Deviation) (*n =* 3).

In our study, the pH sensitive P123-DTX conjugate micelles were added into rat plasma and the amount of released DTX and DTX-L were quantified by high-performance liquid chromatography (HPLC) (Figure 6). Although DTX was linked to P123 by covalent hydrazone bond, both of the pH sensitive micelles formed by conjugates still exhibited a sustained release profile within 10 h. After released for 48 h, the cumulative release amount of DTX (and DTX-L) from conjugate micelles was only about 19.5%. These observations showed that our prepared conjugate micelles had a favorable stability around blood circulation.

**Figure 7:**
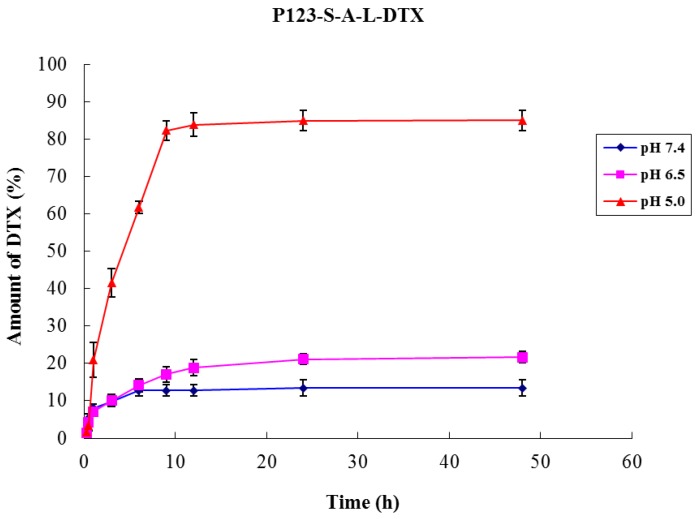
pH-dependent release of DTX from the P123-DTX conjugate micelles. Data are expressed as means ± SD (*n =* 3).

We examined the DTX release from the two P123-DTX conjugate micelles at different pH Values. In order to release the drug at target sites (at mildly acidic pH level), the acid-cleavable hydrazone linkage was introduced to combine DTX and Pluronic P123. The P123-DTX conjugate micelles were incubated for 48 h at pH 5.0, 6.5 and 7.4. The total extent of the released drug was calculated as the sum of DTX and DTX-L using the HPLC method. The results showed that the hydrolysis rates of hydrazone linkage in the conjugate micelles were pH-dependent (Figure 7). As expected, DTX released more efficiently at lower acidic pH (pH 5.0), and the release extents of the conjugate micelles could reached above the 80% (~84.9%) at 48 h. It was near six times higher than the release profile at physiological pH level (pH 7.4), which was only about 13.4% released from the conjugate micelles at 48 h.

## 3. Experimental Section

### 3.1. Materials

Pluronic P123 (P123) was purchased from BASF China Co., Ltd. (Shanghai, China). Docetaxel and Duopafei^®^ were provided by Qilu Pharmaceutical Co., Ltd. (Ji’nan, China). 4-oxopentanoic acid (levulic acid, LEV), adipic dihydrazide (ADH), Succinic anhydride (Suc), 1-ethyl-3-(3-dimethylaminopropyl) carbodiimide hydrochloride (EDC·HCL), *N*-Hydroxysulfosuccinimide sodium salt (Sulfo-NHS), 4-dimethylaminopyridine (DMAP), and pyrene were all obtained from Aladdin^®^ (Shanghai, China). All other chemicals and reagents used were of analytical grade or higher and obtained commercially.

### 3.2. Synthesis of Levulic Acid Derivatives of DTX (DTX-L)

DTX was esterified on 2'-hydroxyl of DTX with LEV to afford the respective ester derivate, designated as DTX-L, with a little difference to the procedure previously reported. Briefly, EDC·HCL (24.5 mg) and LEV (17.6 mg) were dissolved in 2 mL Dichloromethane (DCM) and cooled for 20 min to 4 °C. Then 3 mL DCM solution of DTX (92.5 mg) and DMAP (18.4 mg) was added dropwisely. The reaction was stirred at 4 °C for 8 h. Then DCM was evaporated and ethyl acetate was added to dissolve the product. The organic phase was collected and washed with 10 mL solution of HCl (1%, *w*/*v*) and 10 mL of ultrapure water twice, respectively, to remove DMAP and unreacted succinic anhydride. Magnesium sulfate was added to the organic phase and incubated overnight to remove remained water. The final solution was subjected to silica gel column chromatography for purification.

### 3.3. Synthesis of Hydrazone Contained Derivatives of DTX (DTX-L-A)

The hydrazone contained derivatives of DTX was achieved by the reaction of the DTX-L and adipic dihydrazide (ADH). Briefly, DTX-L (20.6 mg) and ADH (40.2 mg) were dissolved in 3 mL anhydrous methanol (MEOH) under stirring at 60 °C. The reaction was performed for 4 h after addition of acetic acid (AA, 40 µL/mL of reaction mixture). Then the reaction solution was cooled to 25 °C, filtered and recrystallized from methanol. Finally, silica gel column chromatography was used for purification.

### 3.4. Synthesis of Carboxyl Contained Derivatives of Pluronic P123 (P123-S)

To obtain the carboxyl contained derivatives of Pluronic P123, the Succinic anhydride (Suc) was used to react with the hydroxyl group of the Pluronic P123. Briefly, Pluronic P123 (123.5 mg) and Succinic anhydride (45.7 mg) were dissolved in 5 mL DCM, and DMAP (23.9 mg) was added under stirring condition. The reaction was performed for 24 h at 25 °C. Then DCM was evaporated and methanol was added to dissolve the product and finally dialyzed against distilled water (Molecular Weight (MW) cutoff 3500 Da) for 48 h and lyophilized.

### 3.5. Synthesis of Pluronic P123-DTX Conjugate Contained Hydrazone Bond (P123 -DTX)

Pluronic P123-DTX conjugates contained one hydrazone bond were synthesized by the reaction between DTX-L-A and P123-S. Briefly, P123-S (89.5 mg), EDC·HCL (25.5 mg) and Sulfo-NHS (17.8 mg) were dissolved in 3 mL dimethyl sulfoxide (DMSO), and react for 5 h at 4 °C to afford the Sulfo-NHS-S-P123. Subsequently, 4 mL DMSO solution of DTX-L-A (21.7 mg) and DMAP (19.3 mg) was added dropwisely. The reaction was stirred at 25 °C for 24 h. Then the reaction solution was dialyzed against phosphate buffer (pH 7.4) (MW cutoff 3500 Da) for 48 h and lyophilized [24].

### 3.6. Preparation and Characterization of pH Sensitive Pluronic P123-DTX Conjugate Micelles

The pH sensitive P123-DTX conjugate micelles were prepared by a dialysis method: lyophilized P123-DTX conjugates were dissolved in tetrahydrofuran (total concentration 150 mg/mL). The resulting suspension was dialyzed against phosphate buffer (pH 7.4) (MW cutoff 3500 Da) for 36 h.

The weight percentage (wt%) of DTX in the two conjugates was determined using an UV-Vis spectrophotometer based on our previous study [24]. The hydrodynamic particle sizes of these conjugate micelles were evaluated by dynamic light scattering (DLS) (Beckman Coulter Inc., Brea, CA, USA). Transmission electron microscopy (TEM) (JEOL Ltd., Tokyo, Japan) was employed to visualize the morphology of P123-DTX conjugate micelles with the phosphotungstic acid method.

### 3.7. Determination of Critical Micelle Concentration

The critical micelle concentration (CMC) of the pH sensitive conjugates were determined using the fluorescence probe technique as we described previously [24]. Briefly, P123-DTX conjugate micelles solutions with the concentration ranging from 1.0 × 10^−5^ to 6.0 × 10^−4^ M were equilibrated with a fixed concentration of pyrene, 1.0 × 10^−7^ M. At the fixed excitation wavelength of 334 nm, the emission spectras were scanned from 350 to 500 nm. The fluorescence intensity ratios of pyrene at 373 and 383 nm (*I*_373_/*I*_383_, *I*_1_/*I*_3_) were calculated and plotted against the concentration logarithm of the P123-DTX conjugate. The CMC was obtained from the threshold concentration of self-assembled micelles.

### 3.8. Stability of P123-DTX Conjugate Micelles against Mouse Serum

To better determinate the stability of the conjugate micelles, we also detected the drug release situations against the rat plasma. The study were carried out in mouse plasma at 37 °C for up to 48 h according to the method another DTX conjugate mentioned [26] with a little difference. Briefly, 0.5 mL solution of the P123-DTX conjugate micelles and DTX/P123 micelles (all with an equivalent DTX concentration of 700 µg/mL) were measured and added into 2.5 mL of rat plasma at 37 °C. The amounts of released DTX and DTX-L at different time were quantified by reverse-phase high-performance liquid chromatography (RP-HPLC) analysis (Shimadzu Co., Ltd., Kyoto, Japan) on a C18 column with acetonitrile/water (50:50) as eluting solution at a flow rate of 1.0 mL/min.

### 3.9. Assay of in Vitro Drug Release

The *in vitro* release of drug from the P123-DTX conjugate micelles was investigated using the dialysis method we described previously [24]. Briefly, 2 mL of the P123-DTX conjugate micelles solutions (both diluted at an equivalent DTX concentration of ~100 µg/mL) were placed into the pre-swelled dialysis bags (MW 3500 Da) and then dialyzed against 10 mL of phosphate buffer (pH 5.0, 6.5 and 7.4) containing 0.5% Tween 80 at 37 ± 0.5 °C under oscillation at 100 r/min. At selected time intervals, the solutions were sampled. Released DTX and DTX-L was analyzed by the HPLC method.

## 4. Conclusions

We synthesized the pH sensitive Pluronic P123-DTX conjugates by the combination between the Pluronic P123 and the drug docetaxel via acid-cleavage hydrazone bonds. With a low critical micelle concentration (CMC), these pH sensitive P123-DTX conjugates could self-assemble into nano-size polymeric micelles in aqueous solution. The spherical morphology and particle size of the prepared nano-micelles were characterized by transmission electron microscopy (TEM) and dynamic light scattering (DLS), respectively. With the introduction of the covalent hydrazone bonds, these pH sensitive polymeric conjugate micelles exhibited their stability against the rat plasma, and showed a pH dependent drug release behavior. These results showed that our prepared pH sensitive Pluronic P123-DTX conjugate micelles had the potential to balance the stability and drug release. And they might offer a great benefit for drug delivery and controlling the drug release.
